# Top-Down Dysregulation—From ADHD to Emotional Instability

**DOI:** 10.3389/fnbeh.2016.00070

**Published:** 2016-05-23

**Authors:** Predrag Petrovic, F. Xavier Castellanos

**Affiliations:** ^1^Department of Clinical Neuroscience, Karolinska InstitutetStockholm, Sweden; ^2^Department of Child and Adolescent Psychiatry, The Child Study Center at NYU Langone Medical CenterNew York, NY, USA; ^3^Nathan Kline Institute for Psychiatric ResearchOrangeburg, NY, USA

**Keywords:** ADHD, emotional instability, top-down regulation, prefrontal, cingulate

## Abstract

Deficient cognitive top-down executive control has long been hypothesized to underlie inattention and impulsivity in attention-deficit/hyperactivity disorder (ADHD). However, top-down cognitive dysfunction explains a modest proportion of the ADHD phenotype whereas the salience of emotional dysregulation is being noted increasingly. Together, these two types of dysfunction have the potential to account for more of the phenotypic variance in patients diagnosed with ADHD. We develop this idea and suggest that top-down dysregulation constitutes a gradient extending from mostly non-emotional top-down control processes (i.e., “cool” executive functions) to mainly emotional regulatory processes (including “hot” executive functions). While ADHD has been classically linked primarily to the former, conditions involving emotional instability such as borderline and antisocial personality disorder are closer to the other. In this model, emotional subtypes of ADHD are located at intermediate levels of this gradient. Neuroanatomically, gradations in “cool” processing appear to be related to prefrontal dysfunction involving dorsolateral prefrontal cortex (dlPFC) and caudal anterior cingulate cortex (cACC), while “hot” processing entails orbitofrontal cortex and rostral anterior cingulate cortex (rACC). A similar distinction between systems related to non-emotional and emotional processing appears to hold for the basal ganglia (BG) and the neuromodulatory effects of the dopamine system. Overall we suggest that these two systems could be divided according to whether they process non-emotional information related to the exteroceptive environment (associated with “cool” regulatory circuits) or emotional information related to the interoceptive environment (associated with “hot” regulatory circuits). We propose that this framework can integrate ADHD, emotional traits in ADHD, borderline and antisocial personality disorder into a related cluster of mental conditions.

## Introduction

Arguably, altered regulation of information processing represents the core underlying mechanism for many psychiatric disorders. Such aberrations can affect multiple dimensions, including both non-emotional and emotional processes. Currently, some disorders are conceptualized as dysfunction of one or the other of these dimensions. For example, attention-deficit/hyperactivity disorder (ADHD) is defined on the basis of dysfunctional regulation of non-emotional information processing (including inattention, impulsivity and hyperactivity), whereas disorders such as borderline personality disorder (BPD), antisocial personality disorder (ASPD) and conduct disorder (CD) entail symptoms reflecting emotional instability associated with dysfunctional regulation of emotional processes. Other disorders involving emotional instability include intermittent explosive disorder and oppositional defiant disorder (ODD). Traditionally, these psychiatric disorders have been studied separately, reflecting their historical categorical divisions, and the tendency of investigators and disciplines to reify such distinctions. However, these historical distinctions are increasingly appreciated as having impeded understanding of specific disorders in relation to each other. In this Hypothesis and Theory article, we propose that dysregulation in these different dimensions can be incorporated into a unified model.

Although disorders that include persistent emotional dysregulation differ in many symptoms, recent neurocognitive research results suggests common features are involved in emotional dysfunction. These “emotional instability disorders” are characterized by emotional hyper-responsiveness in amygdala and insula, regions involved in shaping emotional responses and experience (Blair, 2010; Rubia, 2011; Blair et al., 2014; Glenn and Raine, 2014; Krause-Utz et al., 2014). Structurally, the gray matter volumes of amygdala and insula are often reduced in such disorders (Blair, 2010; Rubia, 2011; Blair et al., 2014; Glenn and Raine, 2014; Krause-Utz et al., 2014). These observations suggest that a more general approach to study emotional instability may reveal insights into common underlying mechanisms. In this article, we will illustrate our thesis by focusing on ADHD and BPD. Less emphasis will be placed on CD and ASPD since these disorders also include sub-populations with callous-unemotional traits that are associated with decreased behavioral and neural responding to emotional stimuli, potentially confounding the contribution from emotional instability traits (Blair, 2010, 2013; Rubia, 2011; Blair et al., 2014). We believe the literature on the neurobiology of ODD and intermittent explosive disorder is currently insufficient to warrant their inclusion in this illustrative essay, although the approaches we highlight should also be applicable in future studies.

Several reviews and theoretical articles have recently discussed how top-down regulation in ADHD differs in comparison to specific emotional instability disorders including BPD (Sebastian et al., 2014) and CD (Rubia, 2011; Blair et al., 2014). The difference between putative regulatory systems involved in classical ADHD and emotional variants of ADHD has also been noted (Castellanos et al., 2006). Here we focus on the relationship between ADHD and emotional instability disorders in general. Instead of emphasizing their many differences, we seek to place these disorders in a common theoretical framework.

In more detail, we will put forward a model in which ADHD and emotional instability disorders are mechanistically related. We will argue that the fundamental problem in both types of disorders is a similar dysfunctional top-down regulation of information processing, in which the difference between the two types of categorical disorders (ADHD vs. emotional instability disorders) is whether the dysfunctional top-down regulation is associated with emotional (and interoceptive) processing or non-emotional (and exteroceptive) processing (*Hypothesis 1*). Given that the disorders are mechanistically related we also hypothesize that the symptoms associated to one type of dysregulation will be overly represented in patients that have the disorder associated with the other type of dysregulation—even when there is no explicit comorbidity (*Hypothesis 2*). Thus, a dimensional approach would better describe the existing phenotypes than categorical distinctions. Finally, if the underlying mechanisms are similar, treatments proven to be efficacious for one category of disorders should also be efficacious for the other category of disorders (*Hypothesis 3*). This could open new important possibilities for treatment.

We first discuss a dimensional approach to psychiatric disorders, as this is central for understanding the relation between emotional and non-emotional dysregulation in clinical populations. We then take up the relation between non-emotional executive functions (“cool” executive functions) and ADHD. Given that executive functions are fundamentally associated with ADHD, we then review the underlying prefrontal networks in the brain mediating such top-down control and show that these systems are altered in ADHD. A similar review will be done for emotion associated (“hot”) executive functions and emotional regulation, and their relation to emotional instability disorders as well as for “emotional” traits in ADHD. We then discuss how systems mediating emotional and non-emotional top-down regulation (and dysregulation) relate to each other in prefrontal, striatal and dopamine networks. We will also discuss how this stratification between emotional and non-emotional regulation could be discussed in terms of systems related to interoceptive and exteroceptive processing. Finally, we present a model that can incorporate both ADHD and emotional instability disorders based on the relationship between these top-down regulatory systems.

## Categorical and Dimensional Approaches to Psychopathology

In psychiatry, the adoption of the third edition of the Diagnostic and statistical manual of mental disorders (DSM; American Psychiatric Association, 1980) initiated the practice of defining psychiatric disorders as present or absent depending on whether a minimum number of clinical criteria were satisfied. This categorical approach enhanced the reliability of psychiatric diagnoses, but it has not advanced our understanding of underlying mechanisms (Insel et al., 2010; Cuthbert and Insel, 2013). One problem is that many psychiatric symptoms are continuously distributed in the general population. Truncating the range of variation by applying arbitrary cut-points impedes an understanding of underlying mechanisms since it does not mirror the true relationship between symptom levels and neurocognitive levels. Moreover, only focusing on psychiatric disorders excludes data from healthy individuals that are not treated with medication nor show any comorbidities—factors that confound categorically based research. Another problem is that defining disorders categorically based on whether criteria cut-points are met increases heterogeneity. Two patients can differ on nearly every symptom and still receive the same diagnosis. Moreover, in existing categorical diagnostic systems such as the 5th edition of the DSM (DSM-5; American Psychiatric Association, 2013) or the 10th edition of the International classification of diseases (ICD-10; World Health Organization, 2011), a particular diagnosis can be partially defined by opposite symptoms. For example, patients with depression can sleep too much or too little, have increased or decreased appetite, or increased or decreased activity levels. Logically, different underlying mechanisms could mediate these behaviors—although an alternative hypothesis is that both extremes become more likely when a regulatory process is dysfunctional (Klein, 1999). Accordingly, investigators are being urged to focus on specific *fundamental behavioral components* that may be altered in multiple psychiatric disorders, such as attention or emotional regulation as a part of the Research Domain Criteria (RDoC) initiated by the USA National Institute of Mental Health (NIHM; Insel et al., 2010; Cuthbert and Insel, 2013). Variation of such functions in the general population has specifically been identified as a promising way to understand dysfunction in these systems.

By focusing on how information is processed on a systems level, cognitive neuroscience has been successful in describing mechanisms underlying normative human perception and behavior (Gazzaniga, 2014). A major challenge for cognitive neuroscience is to translate such basic knowledge of brain function to psychiatric disorders. From a cognitive neuroscience perspective, specific cognitive processes underlie particular behaviors—here we term those *cognitive core processes*. Building on previously given examples of fundamental behavioral components from the RDoC such as attention and emotional regulation, we define cognitive core processes as the underlying neuronal mechanisms, on a system level, needed to produce a given behavior. There are many different attentional and emotional regulation processes and each process may include multiple aspects. For example, ample data suggest that amygdala modulates visual processing of threat cues (Vuilleumier and Driver, 2007; Vuilleumier, 2015). The specific modulation of amygdala on information processing in visual cortex may then be defined as a cognitive core process. Dysfunctions in these processes underpin the fundamental behavioral components that are coupled to different psychiatric states (Insel et al., 2010; Cuthbert and Insel, 2013). Variability between individuals in different cognitive core processes may underlie behavioral differences among healthy individuals but also clinical symptoms beyond the normative range. As cognitive neuroscience often includes analyses of inter-subject variability in cognitive core processes (Bishop, 2009; Indovina et al., 2011), it is well suited to study variation in fundamental behavioral processes (Insel et al., 2010; Cuthbert and Insel, 2013) related to specific psychiatric symptoms.

**Figure 1:**
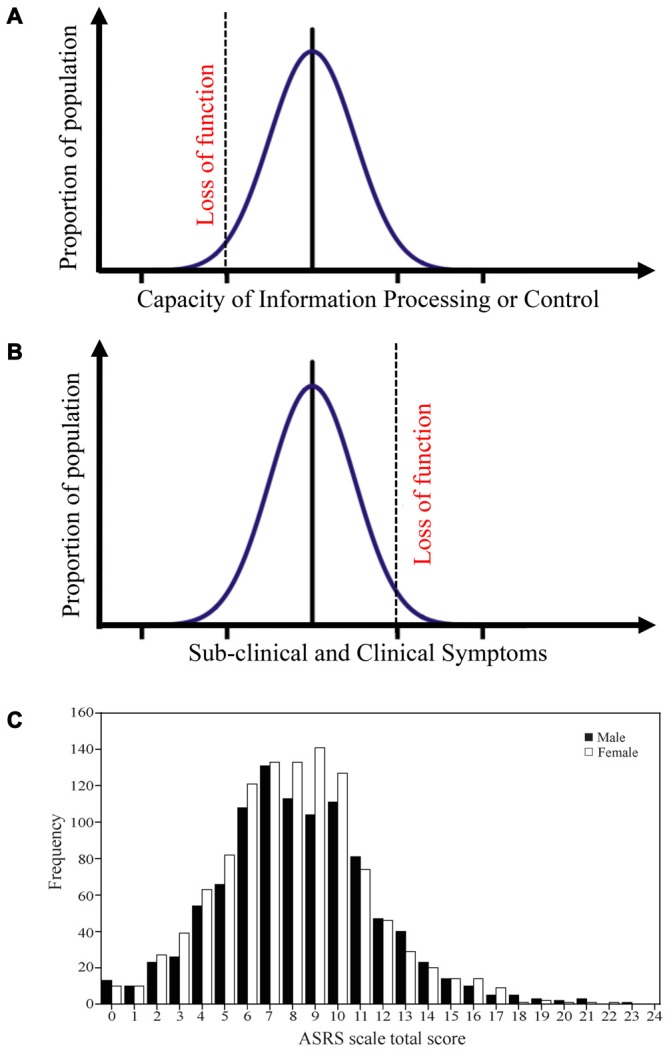
**Distribution of processing capacities and symptoms in the general population. (A)** A model that suggests that different cognitive core capacities in the population often follows a normal distribution. Extremely low cognitive core capacities will induce a functional loss. Such a distribution is for example observed for executive function capacity. **(B)** The model posits that this capacity is mirrored in different behavioral symptoms, and that the transition from sub-clinical to clinical symptoms is defined by functional loss. **(C)** ADHD symptoms in the general population follows such a distribution. Panel **C** is originally from Das et al. (2012) and reprinted with permission from *PLos One*.

Studies adopting a neurocognitive endophenotype approach have often compared patients and healthy next-of-a-kin in specific behaviors and underlying structure/processes that are more present in these groups than in controls (Ersche et al., 2010, 2012, 2013; Morein-Zamir et al., 2013). An alternative approach is to directly study variability in the general population. A fundamental question is then how variation in the capacity to process information relates to clinical symptoms. One hypothesis is that the capacity to carry out cognitive core processes is inversely and directly related to certain psychiatric symptoms. From an information-processing viewpoint, cognitive core process capacities vary in the population from extremely efficient to extremely inefficient, depending on underlying genetic composition, learning history and state variables. This variation is often normally distributed (Figure 1A), e.g., in executive functions (Zelazo et al., 2013). Since cognitive processes form and shape individual behaviors, suboptimal cognitive core capacity can translate into behavioral symptoms. The frequency and intensity of these symptoms will be continuously distributed in the general population—with most below the threshold for clinical significance (Figure 1B). However, increasingly severe symptoms impair functioning, making it difficult for the affected individual to uphold expected social relations, or be occupationally productive. Such loss of functioning is the *sine qua non* of psychiatric disorders. To the extent that psychiatric disorders constitute extremes in variation across the population caused by suboptimal cognitive core process function, dimensional approaches will be a better fit than categorical ones. This has been suggested for several disorders such as ADHD (Figure 1C; Das et al., 2012).

## “Cool” Executive Functions and ADHD

Applying the model described above on dimensional approaches of psychopathology suggests that the worse cognitive capacity an individual possesses, the more symptoms he or she should manifest. Empirically, the general dimensional model seems to hold particularly well for ADHD. For example, in a non-clinical sample of more than 2000 adults, ADHD symptoms were normally distributed (Das et al., 2012). The dimensionality of ADHD symptoms has also been repeatedly observed in patient samples (Levy et al., 1997; Salum et al., 2014). Early models of ADHD posited that ADHD is a disorder of dysfunctional executive functions (e.g., Barkley, 1997). In line with these ideas, ADHD patients as a group tend to perform below average in laboratory tests of executive function capacity (Willcutt et al., 2005). While there is evidence suggesting a dimensionality for both ADHD symptoms and executive function capacity, associating trait-like capacities for executive functions to ADHD symptoms in the general population is not trivial. Namely, test performance can vary across and within individuals as a function of numerous factors including alertness/arousal, motivation, and past experience/exposure. Other clinical conditions such as traumatic brain injury or periodic psychiatric problems (e.g., mood disorders) also may affect executive function performance. Correspondingly, numerous traits may be related to ADHD, only some of which are related to dysfunctional executive functions (Castellanos and Proal, 2012). Finally, different individuals may rate the same behavior differently depending on cultural factors and meta-cognitive capacity. Still, a community study of more than 16,000 children and adolescents provides some support for the dimensionality of ADHD symptoms and their relationship to putative executive function capacity (Crosbie et al., 2013). Specifically, the study reproduced, in a general pediatric population, the normal distribution of ADHD-symptoms (Das et al., 2012). Moreover, rated attentional problems in daily life correlated with putative executive function capacity on the stop signal test. The stop signal test measures capacity to suppress an initiated movement and is frequently used for measuring executive functions in ADHD (Nichols and Waschbusch, 2004; Alderson et al., 2007). The relationship between real life problems and stop signal performance was linear across the entire distribution rather than limited to the children who had been diagnosed with ADHD. Thus, there is evidence that both ADHD symptoms and executive function capacity are normally distributed in the population, and that they are associated with each other. On a more general level this suggests that there is a relation between the capacity of specific *cognitive core processes* and symptom severity in the population extending from healthy individuals to those with frank psychiatric states.

## Neuroatonomy of “Cool” Executive Functions

Since executive functions and ADHD symptoms appear to be linked, understanding the underlying neural mechanisms mediating the cognitive processes should help elucidate how related clinical symptoms emerge. Executive functions may be defined as a set of control mechanisms that regulate non-routine information processing including behavioral suppression, task switching, adaption, or change of strategy (Barkley, 2012; Goldstein and Naglieri, 2014). To distinguish classical executive functions from those related to emotional processes, the former have been termed “cool” executive functions. These functions are dependent on the prefrontal cortex (PFC) although they represent distributed network processes encompassing many different brain regions including the basal ganglia (BG) and brainstem neuromodulatory systems. The circuits subserving executive functions also involve thalamus, parietal cortex and cerebellum. However, for simplicity, we abbreviate these complex circuits by referring primarily to the prefrontal and anterior cingulate cortex (ACC). In part resulting from Barkley’s (1997) suggestion of the primacy of inhibitory capacity in ADHD, many investigators have examined performance on the stop-signal test (Crosbie et al., 2013) and the Stroop test (Lansbergen et al., 2007), both of which involve inhibitory aspects.

**Figure 2:**
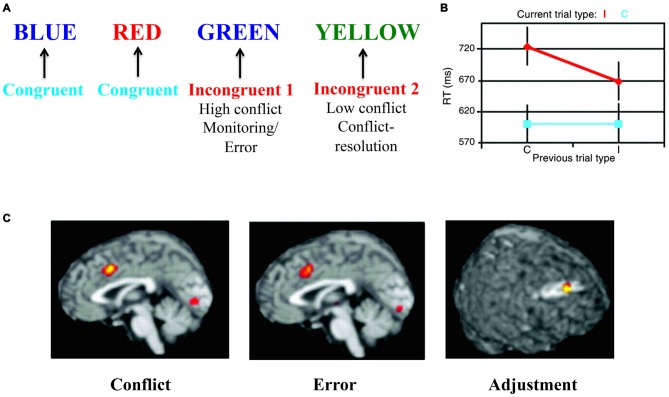
**The Stroop task—as an example of “cool” executive function tasks. (A)** In the Stroop task colored words are presented where the word itself represents a color. The task is to indicate the color of the word but ignore the word meaning. In some conditions both the color and the word are associated with the same color (congruent condition), while in other conditions the color and the word are associated with different colors (incongruent condition). In the incongruent condition there is a conflict between processes associated with color identification/naming and reading. In order to solve the task, this cognitive conflict has to be regulated using top-down control by suppressing the conflicting process and strengthening the processes associated with the correct response. If two incongruent stimuli are presented in a row, the conflict and error signal is somewhat smaller in the second incongruent condition as conflict-resolution (i.e., adjustment) has been engaged (Kerns et al., 2004). **(B)** Behaviorally, response times mirror the cognitive conflict (red vs. blue) and its resolution (smaller response time in the second incongruent condition vs. the first incongruent condition—both in red). **(C)** These different conditions are mirrored in the underlying brain activations of caudal anterior cingulate cortex (cACC) and dorsolateral prefrontal cortex (dlPFC) representing conflict, error and adjustment. Panels **B,C** are originally from Kerns et al. (2004) and reprinted with permission from AAAS.

The Stroop test targets the involvement of executive functions in resolution of a cognitive conflict mediated by an incongruent stimulus (see Figure 2). This test of executive functions is especially interesting for the model presented here because it can also be used in the emotional domain (Egner et al., 2008; Eippert et al., 2009). A seminal article (Kerns et al., 2004) used the Stroop task to decompose different neuronal aspects of executive functions. This study leveraged the conflict resolution that occurs when the same cognitive conflict condition is repeated (see Figure 2A). By presenting two incongruent stimuli in a row, Kerns et al. (2004) differentiated two separate conditions for the same type of incongruent stimulus: (1) when conflict was high and conflict resolution was low (first incongruent stimulus); and (2) when conflict resolution was high and conflict was low (second incongruent stimulus). The first type of stimulus presentation evoked an error signal that could activate prefrontal conflict resolution systems and decrease conflict in the next stimulus presentation. These manipulations were reflected behaviorally in terms of magnitude of response-time cost of incongruency and in neural signals. Specifically, activation in caudal anterior cingulate cortex (cACC), observed in the first incongruent stimuli condition, was associated with conflict and error-signals (see Figure 2C; Kerns et al., 2004) or with initially resolving the conflict (Roelofs et al., 2006; Aarts et al., 2008). Subsequent activation of dorsolateral prefrontal cortex (dlPFC), observed in the second incongruent stimulus condition, was interpreted as reflecting updating the rules used to more effectively solve the conflict (see Figure 2C).

Meta-analyses suggest that similar regions including cACC and dlPFC are also involved in other executive function tasks including spatial interference, stop-signal task, go/no-go task, flanker task and Simon task (Nee et al., 2007; Cieslik et al., 2015). Apart from those regions, parietal cortex is also critically involved in executive function tasks as a part of frontoparietal executive control networks. Interestingly, both ventrolateral PFC (vlPFC) and anterior insula are involved in such executive function tasks (Nee et al., 2007; Whelan et al., 2012; Cieslik et al., 2015)—although they often are assigned to emotional processing systems.

## Neuroanatomy of “Cool” Executive Functions in ADHD

**Figure 3:**
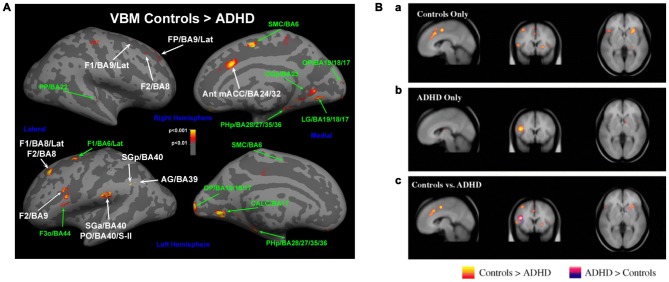
**Altered networks in ADHD associated with “cool” executive functions. (A)** Some studies have suggested a smaller gray matter volume in cACC and dlPFC (e.g., Seidman et al., 2011), and **(B)** diminished activations in these networks during executive function tasks in patients with ADHD compared to controls (Dickstein et al., 2006). Panel **A** is originally from Seidman et al. (2011) and reprinted with permission from Elsevier. Panel **B** is originally from Dickstein et al. (2006) and reprinted with permission from John Wiley & Sons, Ltd.

Dysfunctional executive functions observed in ADHD patients should be mirrored in the underlying structure and function of systems mediating this regulation (Castellanos et al., 2006; Bush, 2010). In line with this idea, maturation of the thickness of the cortex, which follows a normative inverted-U trajectory, was found to be significantly delayed in children with ADHD across nearly the entire cortex, with greatest delays in PFC and ACC (Shaw et al., 2007). In a naturalistic comparison, adolescents taking psychostimulants differed in the rate of change of cortical thickness from those not taking psychostimulants (Shaw et al., 2009), suggesting that medication might ameliorate the delayed development. Thinning in the medial and dlPFC was persistent only in those patients that maintained the full ADHD diagnosis in adulthood (Shaw et al., 2013). An earlier and smaller study found that adult patients with ADHD displayed decreased smaller gray matter volumes in both cACC and dlPFC, although the findings were modest and did not survive full brain correction (Seidman et al., 2011; Figure 3A).

Similarly, activation of these networks is altered during top-down regulatory tasks in ADHD-patients. Meta-analysis of functional imaging studies reveals decreased activation in patients with ADHD in systems involved in executive functions (e.g., the frontoparietal networks) and attention (e.g., ventral attentional network) in task-based studies (Cortese et al., 2012). When studies were restricted to inhibition or attention tasks, ADHD patients showed reduced activation in similar networks (Dickstein et al., 2006; Hart et al., 2013; Figure 3B). While the deficit in inhibition activation was more prominent in right inferior frontal cortex, supplementary motor area (SMA) and ACC, the deficit in attention-activation was more prominent in dlPFC, parietal and cerebellar areas (Hart et al., 2013). Specifically in the Stroop task, dlPFC was less activated for sustained attentional control and the cACC less activated for transient aspects of attentional control (i.e., incongruent trials vs. neutral trials in the incongruent block) in young adults with ADHD vs. controls (Banich et al., 2009).

## “Hot” Executive Functions, Emotional Processing, and Emotional Instability

### Can Emotional Processes be Isolated?

Information processing in the brain is dependent on complex reciprocal interactions between multiple regions in large-scale networks (Mesulam, 1998, 2012; Engel et al., 2001; Dehaene and Changeux, 2011; Siegel et al., 2012). It has therefore been debated whether brain processes associated with “emotion” can be separated from those associated with “cognition” (Pessoa, 2008; Okon-Singer et al., 2015). This question may be rephrased in terms of whether it is possible to separate “emotional” from “non-emotional” processes (and associated regulatory mechanisms), since cognition comprises both emotional and non-emotional information. For example, attention, working-memory and executive-control are not either emotional or non-emotional processes as they operate on both types of information. This point has previously been made for theoretical constructs of attention in distinguishing “cool” executive functions regulating non-emotional processes and “hot” executive functions regulating emotional processes (Zelazo and Mueller, 2002; Kerr and Zelazo, 2004; Castellanos et al., 2006; Rubia, 2011). Therefore, the focus is instead on the possible distinction between emotional processes and non-emotional processes. Clearly, both processes influence each other (Pessoa, 2008; Okon-Singer et al., 2015). In the same vein, any emotional regulatory task will contain non-emotional processes such as holding instructions on line in a working-memory buffer and associated attentional processes.

Nevertheless, the distinction between emotional regulatory systems and non-emotional regulatory systems is useful since there are clinical states in which dysfunction of one pole or the other predominates. Dysregulation is more related to non-emotional processes in classical ADHD while it is more associated with emotional processes in various clinical disorders of emotional instability such as BPD, ASPD or CD. These clinical entities suggest that although emotional and non-emotional processes are both overlapping and interwoven (Pessoa, 2008; Okon-Singer et al., 2015), certain aspects of the involved networks may be more related to emotional or non-emotional dysregulation, respectively. Therefore, to understand the specificity of these disorders, components that are more associated with emotional regulation and non-emotional regulation need to be identified.

Large numbers of emotional regulatory processes have been described (Hartley and Phelps, 2010; Gyurak et al., 2011; Ochsner et al., 2012). While some directly modulate an emotional experience (Ochsner et al., 2012), others are subtler and regulate emotional processes to accomplish a cognitive task (Gyurak et al., 2011), i.e., “hot” executive functions, or are involved in extinction (Schiller and Delgado, 2010; Milad and Quirk, 2012). While processes that regulate emotions are often voluntary and therefore explicit, attentional processes are often automatic and implicit. Here we focus on two fundamental types of emotional regulatory processes that represent different types of top-down control: implicit attentional and explicit cognitive reappraisal processes (Gyurak et al., 2011; Ochsner et al., 2012).

### Implicit Attentional Control of Emotional Processes

The attentional dimension in voluntary regulation of emotional experiences has been extensively discussed (Ochsner et al., 2012). However, for the present purpose automatic implicit attentional mechanisms in strictly defined executive function tasks (Gyurak et al., 2011) may be even more interesting since they have the potential to separate emotional from non-emotional components.

**Figure 4:**
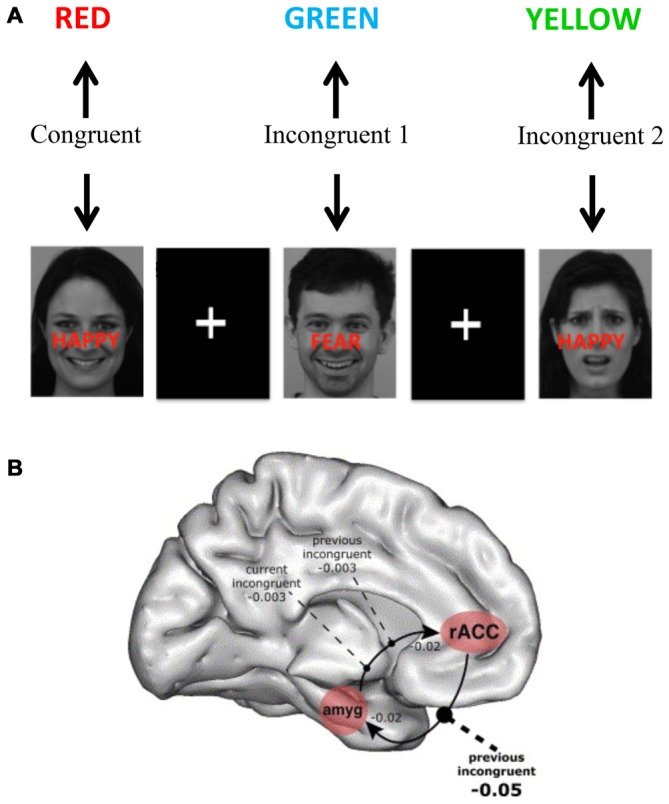
**Brain systems underlying the emotional Stroop task. (A)** The emotional Stroop task has similar features as the original Stroop task including congruent stimuli (without conflict between different processes) and incongruent stimuli (with a conflict between different processes)—but also includes an emotional dimension that needs to be controlled. In the presented example above faces were used from the karolinska directed emotional faces (KDEF; Lundqvist et al., 1998). **(B)** Using a similar version of emotional Stroop task Etkin et al. (2006) showed that higher conflicts increased amygdala involvement and conflict resolution was associated with rostral anterior cingulate cortex (rACC) activation and negative influence on amygdala activity. Panel **B** is orginally from Etkin et al. (2006) and reprinted with permission from Elsevier.

Building on the model of decomposing sub-components of executive functions in the Stroop task (Kerns et al., 2004), Etkin et al. (2006) constructed an emotional Stroop task in which affective facial expressions were displayed with overlaid congruent or incongruent words expressing affects (Figure 4). The task was to report the facial affect and ignore the overlaid words. This yielded both congruent stimuli (in which the facial expression and the word corresponded to the same emotion) and incongruent stimuli incorporating a conflicting process (in which the affective facial expression and emotion word differed). As in the non-emotional version (Kerns et al., 2004), this task could separate an incongruent stimulus in which conflict remained high (first incongruent stimulus) from an incongruent stimulus in which the conflict level was smaller due to conflict resolution (second incongruent stimulus). There was a reaction time increase for the incongruent condition as compared with the congruent condition, indicating that a conflict was successfully induced. Importantly, this increase was smaller for the second incongruent condition compared with the first incongruent condition, indicating conflict resolution. On a neuronal level, amygdala activation was observed in the high conflict condition reflecting increased influence of the non-relevant emotional incongruent stimuli. However, amygdala activity decreased and activity in rostral ACC (rACC) increased in the low conflict (repeated) incongruent condition. Path-analysis indicated that rACC directly suppressed amygdala activity in this condition. Thus, this study suggests that rACC influences amygdala processing to solve an executive function task with emotional content.

A potential confound in the above study was that non-emotional regulation was not controlled for. Therefore, regulatory mechanisms that were specific to emotional regulation could not be differentiated from mechanisms that are shared with non-emotional regulatory processes. A later study by the same group (Egner et al., 2008) sought to distinguish these two dimensions by presenting the same stimuli in two different tasks, differing in emotional intensity. One task was as described above, and the other involved labeling sex with congruent or incongruent words (i.e., “male” or “female”) using the same set of pictures. The original findings were reproduced and survived controlling for the non-emotional regulatory processes. It may be argued that the control for non-emotional regulation was not perfect since both tasks contained affective facial expressions. However, this tradeoff is inevitable since using different types of stimuli in the non-emotional condition would have confounded the task. In sum, this study represents an innovative example of how to control for non-emotional aspects of top-down regulation in automatic attentional tasks.

Other studies on affective interferences in the Stroop test and comparable tasks (Whalen et al., 1998; Ochsner et al., 2009; Rahm et al., 2013) also support the conclusion that similar attentional processes take place in the emotional domain as in the non-emotional domain. The studies converge in highlighting the rACC as specifically involved in emotional executive function tasks, while the cACC is specifically involved in related non-emotional executive function tasks. These findings are consistent with the distinction between “cool” and “hot” executive function processes and their relative regional specificity (Zelazo and Mueller, 2002; Kerr and Zelazo, 2004; Castellanos et al., 2006; Rubia, 2011).

### Explicit Regulation of Emotional Processes

Arguably, the cognitive reappraisal task is one of the most frequently used experimental methods to study emotional regulation (Ochsner et al., 2012; Buhle et al., 2014). Cognitive reappraisal tasks differ substantially from Stroop tasks. While Stroop tasks entail a mechanism that implicitly deals with demanding and fast interference that needs to be resolved on-line for the task to be performed, cognitive reappraisal of emotional stimuli involves an explicit in-depth regulation of information processing. In cognitive reappraisal of emotional stimuli, the task is to change the emotional interpretation of a stimulus. In this way it is more explicit and proactive than Stroop tasks. A task can involve reinterpreting the meaning of emotional pictures from negative to neutral or positive valence. For example, the face of a crying woman, which is automatically interpreted as an emotionally negative picture, can be reinterpreted as a woman who is crying from happiness over seeing her son again after a year. Thus, instead of controlling distracting emotional processes (as in an emotional Stroop task) the reappraisal task actually changes the rules for the emotional interpretation of the world. It requires active assignment of different emotional meaning to stimuli.

The inherent problem of cognitive reappraisal tasks is the difficulty of obtaining behavioral measures since these tasks rely on subjective reports. Moreover, subjects have little insight into what they do during the task. Nevertheless, this has been a popular approach to study emotional regulation since it can dramatically change how we experience the world and it is theoretically close to various cognitive therapies. Cognitive reappraisal tasks have been found to modulate the processing of emotional stimuli in regions such as amygdala and the ventral striatum (Buhle et al., 2014).

**Figure 5:**
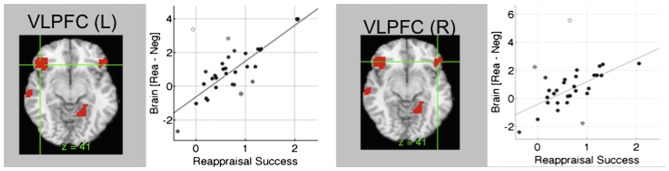
**Brain systems underlying cognitive re-appraisal.** In cognitive reappraisal studies, typically an emotional picture is presented and the subjects are given a task to explicitly down- or up-regulate the emotional content. In conditions where unpleasant pictures are re-interpreted as more positive, amygdala activity is down-regulated and a network of regions is activated including lateral orbitofrontal cortex (lOFC). lOFC activity correlates closely with re-appraisal success during cognitive re-appraisal (Wager et al., 2008). Figure is originally from Wager et al. (2008) and reprinted with permission from Elsevier.

The emotional Stroop task regularly shows prefrontal activations both in dlPFC and lateral orbitofrontal cortex (lOFC)/vlPFC (Eippert et al., 2007; Wager et al., 2008; Kanske et al., 2011; Golkar et al., 2012; Buhle et al., 2014), although other regions (including the dorsal ACC and the dorsomedial PFC) seem to support other components of the task (Ochsner et al., 2012). It has been suggested that dlPFC is involved in general selective attention and working memory, while the lOFC/vlPFC seems to be important for selecting a goal-appropriate reappraisal (Ochsner et al., 2012). Thus, theoretically the lOFC/vlPFC should be more specifically involved in emotional regulatory components of the task. In a few studies, attempts have been made to tease apart these components (Wager et al., 2008; Golkar et al., 2012). In one of the studies (Wager et al., 2008), when the rated success of emotional regulation was regressed on the cognitive reappraisal of emotion contrast, the dlPFC contribution was smaller and that of the lOFC/vlPFC larger than in the standard subtraction analysis (Figure 5). Thus a measurement better reflecting actual emotional regulation was more tightly coupled to lOFC/vlPFC than dlPFC. In another study (Golkar et al., 2012), a neutral control state was introduced which contained non-emotional aspects of the task, and the interaction revealed more specific activation also in the lOFC/vlPFC. Both these studies are examples that suggest that it is possible to partially separate emotional from non-emotional components in the cognitive re-appraisal task and that indicate greater involvement of the lOFC/vlPFC in the emotional component. Thus, as for rACC there seems to be specificity for lOFC in emotional regulation. This also suggests a possible role for this region in disorders involving emotional dysregulation.

### A Role for rACC in Regulation of Emotion and Pain

Previously we have suggested the importance of rACC in implicit attentional regulation in the emotional domain (such as “hot” executive functions). However, a range of other studies suggests a general role for the ACC in emotional regulation. Importantly, although the rACC and cACC differ in their involvement in attentional tasks—as has long been suggested (Bush et al., 2000)—there is little support for a general division between an emotional and a non-emotional cingulate (Etkin et al., 2011; Okon-Singer et al., 2015). In fact, some parts of cACC are highly involved in emotional processes that include negative valence such as pain unpleasantness (Vogt et al., 1993), social exclusion (Eisenberger, 2012) and fear potentiation and appraisal (Etkin et al., 2011; Milad and Quirk, 2012). The cACC is also implicated in behaviors associated with emotional or painful situations and has been viewed as an emotional motor output region (Craig, 2009; Perini et al., 2013). Other influential theories suggest that this part of the cACC has a common role of linking reinforcers to motor centers responsible for expressing negative affect and executing goal-directed behaviors (Shackman et al., 2011) and thereby performing similar fundamental processes for both pain and attention. However, the anatomical and functional relation between ACC involvement in processing of pain/emotion and non-emotional attentional processes is yet to be established.

**Figure 6:**
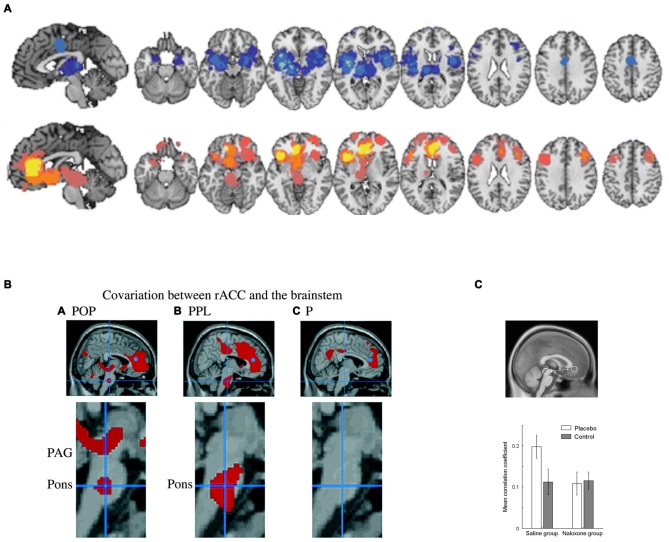
**Brain systems underlying the placebo effect. (A)** Meta-analysis indicating brain networks showing suppressed activity (blue) and increased activity (yellow/orange) related to placebo analgesia (PPL; Atlas and Wager, 2014). Regions showing increased activity include ventromedial prefrontal cortex (vmPFC)/rACC and lOFC. Note that caudal ACC (cACC) decreases in activity after placebo treatment in association with decreased pain unpleasantness. **(B)** Studies also show that PPL involves opioid dependent interactions between rACC and the brainstem. For example, functional connectivity between rACC (blue dot) and the brainstem was observed in opioid analgesia (POP) and in PPL but not in untreated pain (P; Petrovic et al., 2002). **(C)** This connectivity has been shown to be opioid dependent (Eippert et al., 2009). Panel **A** is originally from Atlas and Wager (2014) and reprinted with permission from Springer. Panel **B** is originally from Petrovic et al. (2002) and reprinted with permission from AAAS. Panel **C** is originally from Eippert et al. (2009) and reprinted with permission from Elsevier.

Studies on placebo analgesia (Petrovic et al., 2002; Wager et al., 2004, 2007; Zubieta et al., 2005; Bingel et al., 2006; Eippert et al., 2009; Wager and Atlas, 2015) and emotional placebo (Petrovic et al., 2005; Ellingsen et al., 2013) have also suggested the rACC is involved in top-down regulation of pain and emotion (see Figure 6A). Importantly, this activation remained when controlling for non-emotional attentional aspects of treatment either through interaction or correlation analyses (Petrovic et al., 2002, 2005). The high concentration of opioid receptors expressed in rACC (Vogt et al., 1993; Fields, 2004) has been hypothesized to relate to attentional mechanisms that can suppress pain processing (Petrovic et al., 2002), a suggestion that has received experimental support (Zubieta et al., 2005; Wager et al., 2007; Eippert et al., 2009). In more general terms, specific neuromodulatory systems in the rACC have been suggested to be involved in conflict resolution in the pain or emotion domains when expectations do not match processing of sensory input (Petrovic et al., 2002). The known anatomical connectivity between opioid systems in rACC and the periaqueductal gray (PAG; Vogt et al., 1993) in turn suggests that such modulation acts through regulating opioid systems in the PAG (Petrovic et al., 2002). This was suggested by functional connectivity between rACC and PAG that was opioid and placebo specific (Petrovic et al., 2002) as well as opioid dependent (Eippert et al., 2009; see Figures 6B,C). More specific opioid connectivity between these structures was also shown (Wager et al., 2007). Apart from the PAG, other regions such as amygdala and ventral striatum are likely involved in the placebo effect and may be directly controlled by the rACC (Petrovic et al., 2005; Zubieta et al., 2005; Bingel et al., 2006; Wager et al., 2007; Scott et al., 2008; Eippert et al., 2009; Ellingsen et al., 2013). Thus, studies of the placebo effect suggest that rACC is in a position to regulate emotion and pain processes using specific underlying neuromodulatory systems such as the opioid system. Other neuromodulatory systems may also be involved although this is still unknown. Note that more caudal parts of the ACC process pain *per se* (Petrovic et al., 2002), in line with the idea that cACC is involved in processing the unpleasant aspects of pain (Vogt et al., 1993).

**Figure 7:**
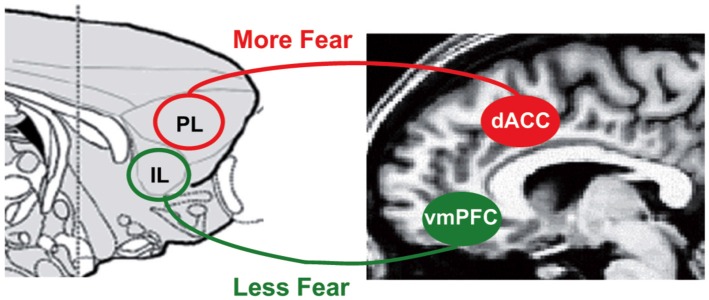
**Brain systems underlying fear conditioning.** In fear conditioning, vmPFC in humans and infralimbic regions (IL) in rodents is associated with suppressed conditioned fear while cACC (dACC here) in humans and prelimbic regions (PL) in rodents is associated with increased conditioned fear through modulation of amygdala (Milad and Quirk, 2012). Figure is originally published by Milad and Quirk (2012) and reprinted with permission from Annual Reviews.

A closely related domain of research on conditioning and extinction in both rodents and humans also suggests that ventromedial PFC (vmPFC), an area neighboring rACC, supports extinction while cACC supports increased aversive processing (Milad and Quirk, 2012; see Figure 7). The vmPFC is thought to modulate amygdala processing of conditioned fear by suppressing fear responses through a set of amygdala neurons in the intercalated region between the basolateral and central amygdala nucleus (Milad and Quirk, 2012). Different strategies to modify conditioned fear, i.e., extinction, reversal and voluntary regulation of fear, have been shown to activate similar regions in vmPFC/rACC (Schiller and Delgado, 2010). In other words, this line of research mimics placebo research in assigning similar regions in the brain an emotional regulatory function on emotional processes in subcortical regions such as amygdala.

Another line of research has suggested that rACC and the neighboring vmPFC are important in processing the subjective value of rewards (Kable and Glimcher, 2007), which may also be associated with opioid activation (Petrovic et al., 2008). In the neuroeconomics literature, overlapping activity often centered on neighboring vmPFC is thought to integrate anticipated values and costs (from regions such as ventral striatum and amygdala) associated with the different options being converted into a single quantity to guide behavior (Ruff and Fehr, 2014).

In summary, converging lines of evidence (including research on emotional executive functions, placebo research and research on extinction of conditioned fear) suggest that rACC and neighboring vmPFC are specifically involved in emotional regulation of lower-level structures including amygdala, PAG and ventral striatum. This effect seems to be specific for emotion/pain processes and supports the idea of a system sub-specialized for “hot” executive functions and emotional regulation.

### A Role for OFC in Regulation of Emotion and Pain

Above we have suggested that OFC has an important role in explicit emotional regulation such as cognitive re-appraisal, in which subjects are asked to modulate the emotions associated with a picture (see above). How does this relate to the general function of OFC? The OFC comprises a complex set of regions that may be differentiated along medial-lateral and anterior-posterior gradients (Ongur and Price, 2000; Ongur et al., 2003; Kringelbach and Rolls, 2004; Kringelbach, 2005). Although the OFC receives input from many sensory modalities and may be the most polymodal region in the cortex (Kringelbach and Rolls, 2004), it is notable that many OFC inputs are interoceptive. Thus, the interoceptive needs of the subject are mapped in OFC where they may interact with reward signals and reinforcers from the exteroceptive world. While more primitive unimodal signals seem to be processed posteriorly, more abstract multimodal signals and reinforcers seem to be processed anteriorly (Kringelbach and Rolls, 2004). A lateral orbital network receives inputs from different sensory modalities and a medial network includes regions in ACC and ventro/dorsomedial prefrontal systems and has been suggested to be an important cortical output for visceromotor structures (Ongur and Price, 2000). The medial-lateral distinction has also been observed in human functional imaging of reward processing in which positive feedback and value are mapped to medial OFC while negative feedback is mapped to lOFC (O’Doherty et al., 2001). These results are in line with findings that lOFC represents aversive error-signals (Seymour et al., 2005).

Recent models of a general OFC function emphasize involvement in constructing expected values based on multimodal inputs (including current internal states) that drive behavior (Schoenbaum and Roesch, 2005; Murray et al., 2007; Schoenbaum et al., 2009; Rudebeck and Murray, 2014). In functional imaging, the association between OFC and complex expected values is illustrated in selective reward devaluation (Small et al., 2001; Gottfried et al., 2003; Kringelbach et al., 2003) and context dependent processing of subjective values (de Araujo et al., 2005; Plassmann et al., 2008).

While the studies above suggest that subjective experience of stimuli may be driven by expectation systems, this has been a principal focus in research on the placebo effect. The lOFC has been activated in several placebo treatment studies (often in co-activation with rACC) as indicated by meta-analysis (Atlas and Wager, 2014; Wager and Atlas, 2015; Figure 6A) including placebo analgesia (Petrovic et al., 2002; Lieberman et al., 2004; Wager et al., 2004) and emotional placebo (Petrovic et al., 2005). Interestingly, the lOFC was specifically activated during placebo analgesia but not during opioid-induced analgesia (Petrovic et al., 2002, 2010) suggesting that some cognitive mechanisms are important for the placebo effect but not for opioid-mediated analgesia. Activation in lOFC is believed to represent treatment expectation and the related error-signal between treatment expectation and incoming nociceptive signal (Petrovic et al., 2010) in line with the idea that predictions about different outcomes drive the placebo response (Buchel et al., 2014). Such expectation related processes in the lOFC have been suggested to drive pain and emotion regulatory signals in rACC and are in line with placebo-specific functional connectivity between these regions (Petrovic et al., 2010).

The involvement of lOFC in cognitive reappraisal and placebo can be understood in light of theoretical models of OFC function (Schoenbaum and Roesch, 2005; Murray et al., 2007; Schoenbaum et al., 2009; Rudebeck and Murray, 2014). In cognitive reappraisal, subjects are instructed to shift their expectation about emotional stimuli, which resembles the expectation manipulation performed in response to placebo. The change of expectations in both paradigms involves assigning new values to emotional stimuli by OFC.

### “Hot” Executive Functions and Emotional Regulation in Emotional Instability

The need for understanding the specific processes underlying emotional regulation is driven by the clinical symptoms often observed in emotional instability disorders such as BPD, ASPD and CD. Although all these disorders have a high rate of comorbidity with ADHD and with ADHD symptoms, they are unquestionably different from classical DSM-5 ADHD. Symptoms involving frequent variability in experiencing intense emotional states and emotion related behaviors are fundamental to emotional instability disorders but not a necessary component of classic ADHD. A central question is therefore whether networks that are more specifically involved in emotional regulation also are more specifically dysfunctional in these disorders. Given the specific involvement of rACC and lOFC in emotional regulation (as outlined above) it may be questioned whether these regions also differ in structure and function in conditions characterized by emotional instability.

**Figure 8:**
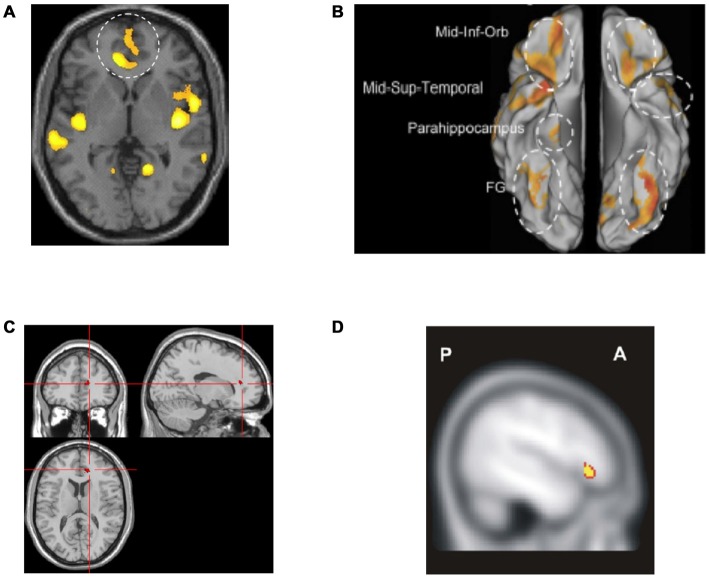
**Altered networks in borderline personality disorder (BPD) associated with “hot” executive functions and emotional regulation. (A,B)** Previous research has suggested smaller gray matter volume in rACC and lOFC in patients with BPD (Soloff et al., 2008, 2012). **(C)** Attenuated activity has been observed in rACC during emotional Stroop (Wingenfeld et al., 2009) in BPD as compared to controls. **(D)** Moreover, lower lOFC activity has been observed during cognitive re-appraisal in BPD as compared to controls (Schulze et al., 2011). Panel **A** is originally from Soloff et al. (2008) and reprinted with permission from Elsevier. Panel **B** is originally from Soloff et al. (2012) and reprinted with permission from Elsevier. Panel **C** is originally from Wingenfeld et al. (2009) and reprinted with permission from Elsevier. Panel **D** is originally from Schulze et al. (2011) and reprinted with permission from Elsevier.

Several studies of brain morphometry have been conducted in emotional instability disorders including BPD, ASPD and CD. Although, they represent initial steps in understanding the underlying pathophysiology, there are two major problems with many of these studies. The first is that most studies have been underpowered, with few patients and controls. The second is that ADHD has not been considered. Nevertheless, two relatively well-powered volumetric studies of BPD (Soloff et al., 2008, 2012) found smaller gray matter volume in rACC and lOFC (Figures 8A,B). Smaller lOFC volume was specifically observed in suicide attempters and even more so in high lethality suicide attempters (Soloff et al., 2012). Similar structural abnormalities have also been observed in other emotional instability disorders such as ASPD (Yang and Raine, 2009). However, such findings have not regularly been observed in morphometric studies of ADHD—in fact the opposite has been observed (Seidman et al., 2011)—as discussed below. The volume of other regions suggested to be involved in emotional processing in BPD, such as amygdala and insula, was also smaller (Soloff et al., 2008, 2012).

Although structural findings can be suggestive, ultimately, the functionality of regions such as rACC and lOFC in tasks involving emotional regulation must be addressed to better understand emotional instability. Few studies have been published so far that directly test “hot” executive functions and emotional regulation in emotional instability disorders. However, some studies suggest a lower activation of regulatory networks in such disorders. Using a verbal emotional Stroop task, Wingenfeld et al. (2009) observed that BPD patients had overall slower reaction times compared to healthy controls but no increased slowing with emotional interference. However, the BPD patients were not able to recruit rACC when they needed to control for negative words (see Figure 8C). Another recent study showed that BPD patients that dissociated were more prone to show greater emotional interference (Winter et al., 2015), although no differences were observed in rACC using a verbal emotional Stroop task. One reason may be that the interference in the verbal emotional Stroop task that was used was too weak, as the controls showed neither a behavioral interference effect nor any rACC activity. By contrast, this was readily observed in Stroop tasks using affective faces (Etkin et al., 2006; Egner et al., 2008). Several other studies using tasks involving emotional conflict control have also shown deficient activations of the subgenual ACC and rACC in patients with BPD (Silbersweig et al., 2007; Enzi et al., 2013; Holtmann et al., 2013; Jacob et al., 2013). Interestingly, in most studies involving patients with BPD in which an emotional conflict must be suppressed, increased activity in amygdala or insula (compared with controls) has been observed, indicating unresolved or heightened emotional conflict (Silbersweig et al., 2007; Krause-Utz et al., 2012; Holtmann et al., 2013; Jacob et al., 2013; Prehn et al., 2013)—but see Smoski et al. (2011). In line with these findings a behavioral study has shown that patients with CD have an impaired Stroop performance under distressing emotional stimulation but no difference under neutral emotional stimulation as compared to controls (Euler et al., 2014). Thus this generalizes the above findings for other emotional instability disorders.

Testing more complex emotional regulation targeting the OFC with cognitive reappraisal of emotional pictures, BPD patients have been found to activate lOFC to a lesser extent than controls while they activated amygdala and insula more (Schulze et al., 2011; Figure 8D). A study of cognitive reappraisal of a script-driven emotional induction did not show differences in lOFC but found lower activations in rACC during up- and down-regulation in BPD subjects compared to controls (Lang et al., 2012).

Although the studies above have not corrected for non-emotional ADHD symptoms nor isolated emotion-specific components in the emotional Stroop-task or during cognitive reappraisal, they indicate that rACC and lOFC appear to be activated to a lesser degree during “hot” executive functions and complex emotional regulation, and are structurally smaller in BPD, the paradigmatic emotional instability disorder.

### Emotional ADHD and “Hot” Executive Functions

ADHD as defined since DSM-III has considered emotional dysregulation an associated feature, rather than a core component. Instead, ADHD has been considered by many to be synonymous with a disorder of executive function. However, as mentioned previously, laboratory measures of executive function are only moderately correlated with ADHD symptoms and they correspond poorly to measures of real world impairment. Interestingly, the effects of stimulant medications impact laboratory measures of executive function less than rated symptoms (Coghill et al., 2014b; Baroni and Castellanos, 2015). Rather, the main effects seem to be on subjective factors such as motivation. Along these lines, phenomenological studies increasingly point to the profoundly impairing effects of emotional dysregulation among many adolescents and adults with ADHD (Castellanos et al., 2006; Thorell, 2007; Yu et al., 2015). In line with this reasoning, including tests on delay aversion and temporal discounting along with standard executive function tests reveals abnormalities in a large majority of ADHD patients (Castellanos et al., 2006; Thorell, 2007; Yu et al., 2015). These tests show that many subjects with ADHD more often chose a smaller reward immediately than a larger reward later in time as compared to controls. Thus, they indicate that altered reward processing and behavior is another important aspect of ADHD rather than only dysfunctional executive functions. These observations have formed the basis for the notion that the neurobiology of ADHD entails at least two pathways, one related to motivation, emotion and reward, and the other focused on dysregulation of action and thought resulting from poor inhibitory control (Sonuga-Barke, 2002; Thorell, 2007; Coghill et al., 2014a).

Few functional imaging studies have addressed the difference between “non-emotional” and “emotional” ADHD traits. However, we note that one study observed that the strength of functional connectivity (magnitude of correlation in spontaneous activity) between amygdala and rACC and between amygdala and posterior insula were significantly correlated with parent ratings of emotional dysregulation in children with ADHD (Hulvershorn et al., 2014)—suggesting that emotional traits in ADHD may influence information processing. Studies of this type are beginning to link emotional dysregulation and brain circuits in the context of ADHD.

## The Relation Between Emotional and Non-Emotional Regulatory Systems

In summary, clinical dysfunctions of top-down regulatory networks can be associated to non-emotional control such as in classic DSM-5 ADHD or to emotional control such as in BPD. We propose that this distinction is tenable because certain regulatory components are more linked to one of the two dimensions although emotional and non-emotional processes highly overlap in the brain. As the cognitive core process capacity of these regulatory systems varies in the population, symptoms associated with both non-emotional dysregulation and emotional dysregulation can be found along a continuum—often normally distributed. This suggests that some clinical states could therefore better be described as extremes on this continuum than as categorically defined disorders.

One important question that arises in this comparison is how dysregulation of emotional processes relates to dysregulation of non-emotional processes. It could be argued that the underlying systems are completely independent, and therefore separate in function and dysfunction. In this case there should not be any increased comorbidity between related clinical disorders (as compared to other disorders) nor an increased risk to have dysregulation in another dimension as compared to healthy subjects. However, this is not the case. The comorbidity between ADHD and the different emotional instability disorders is remarkably large. For example, 42% comorbidity between ADHD and BPD was reported in adolescence and 16% in adulthood in a BPD sample (Philipsen et al., 2008) while the prevalence of BPD in a cohort of 81 patients with ADHD was 37% (Anckarsater et al., 2006). Up to 65% of men with ASPD were reported to present comorbid ADHD (Semiz et al., 2008). Comorbidity among ADHD and CD is substantial (Rubia, 2011). The clinical state of emotional ADHD (Castellanos et al., 2006) also suggests a link between non-emotional and emotional dysregulation. Moreover, ADHD is genetically related to emotional instability disorders such as BPD (Distel et al., 2011). Therefore, the emerging picture suggests both common overlapping mechanisms and specific non-overlapping mechanisms.

**Figure 9:**
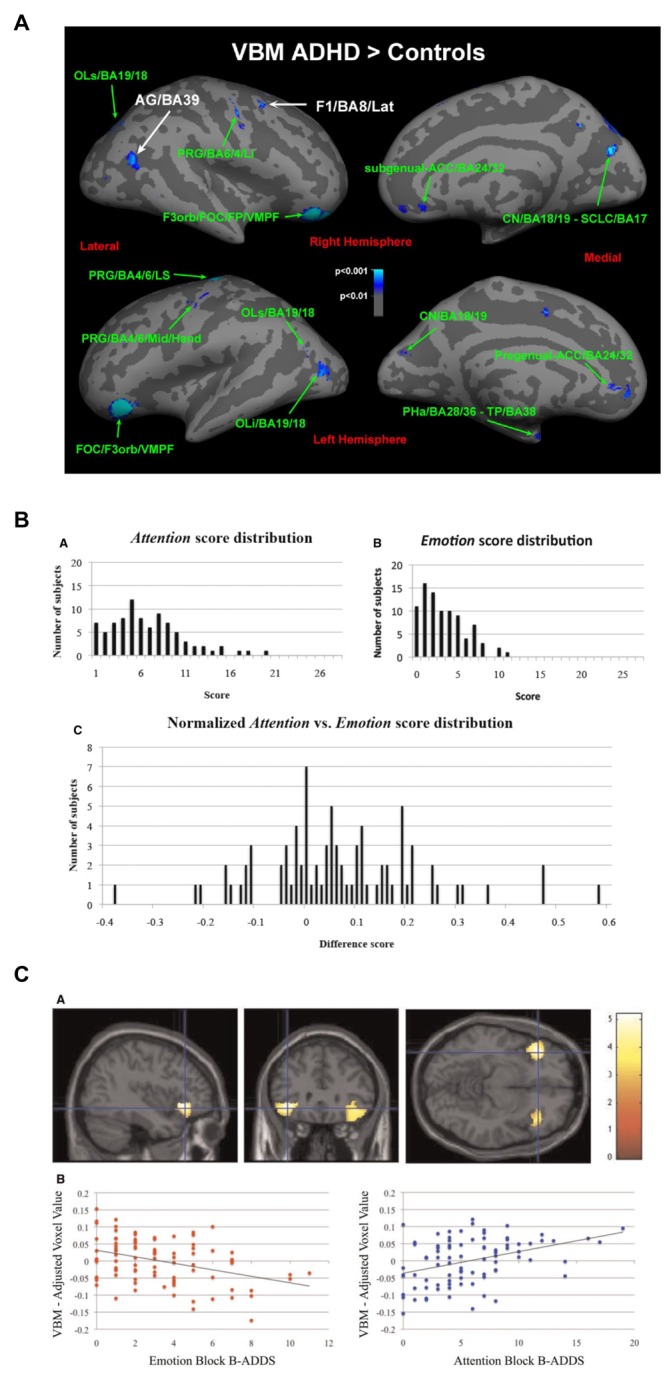
**Relation between networks mediating emotional and non-emotional top-down regulation. (A)** Patients with ADHD without emotional dysregulation disorders showed larger gray matter volume in lOFC and rACC compared with controls (Seidman et al., 2011). **(B)** Variability observed when healthy subjects rate sub-clinical symptoms associated with non-emotional attentional dysregulation and emotional dysregulation (Petrovic et al., 2015) using the Brown Attention-Deficit Disorder Scales (Brown, 1996). **(C)** Gray matter volume in lOFC was negatively correlated with symptoms related to emotional dysregulation but positively correlated with symptoms related to non-emotional attentional dysregulation (Petrovic et al., 2015). Panel **A** is originally from Seidman et al. (2011) and reprinted with permission from Elsevier. Panels **B,C** are originally from Petrovic et al. (2015) and reprinted with permission from Oxford University Press.

However, although research suggests a strong association between non-emotional and emotional regulation, recent brain imaging results suggest that the relation is complex. In a reasonably large study on ADHD that specifically excluded patients with affective problems (Seidman et al., 2011) somewhat smaller gray matter volumes were found in dlPFC and cACC as predicted. An exploratory analysis also found tentative evidence of larger gray matter volumes bilaterally in lOFC and rACC in the ADHD group than in the healthy controls (Figure 9A). By contrast, well-powered studies of BPD (Soloff et al., 2008, 2012) showed smaller gray matter volumes in rACC and lOFC. Thus, the opposite picture emerges for gray matter volume in these regions in classical ADHD (where the affective component is small) compared to BPD.

These results were partially replicated in a recent study on emotional dysregulation in 87 healthy subjects (Petrovic et al., 2015; see Figures 9B,C). In this study, symptoms related to emotional dysregulation correlated negatively with gray matter volume in lOFC bilaterally, while symptoms related to non-emotional attentional dysregulation (i.e., classical ADHD-like symptoms) were positively correlated with gray matter volume in the same region. Thus, it seems that although emotional and non-emotional dysregulation are positively correlated, these dimensions can be inversely related to brain volume in regions that are more involved in non-emotional regulation (such as dlPFC and cACC) and regions that are more involved in emotional regulation (such as lOFC and rACC).

What underlies this puzzling relation between the two regulatory systems is not known. However, these top-down modulatory regions have shown opposite activations during specific attentional tasks (Simpson et al., 2000; Dolcos and McCarthy, 2006; Dolcos et al., 2011)—suggesting mutual active suppression. Moreover, some of these regions such as rACC and cACC tend to correspond to distinct resting state networks that can exhibit anticorrelations during resting state scans (Fox et al., 2005; Fransson, 2005). Although such negative correlations cannot be interpreted as implying mutual inhibition, these robust anti-phase patterns do suggest interactions between emotional and non-emotional regulatory systems in general. We speculate that dysfunction in one regulatory system (e.g., “non-emotional”) could promote the other regulatory system (e.g., “emotional”) to develop and compensate for the dysfunctional system. This imbalance in development would further inhibit the other system leading to even more protracted development. It would be interesting to study whether larger lOFC/rACC gray matter volumes also suggest better emotional regulation after controlling for the non-emotional components in an experimental task.

The findings that emotional dysregulation and non-emotional dysregulation may be inversely related to brain volume and activation patterns in prefrontal regulatory networks suggest that both dimensions should be assessed simultaneously. Thus, endophenotype dimensions relating to emotional dysregulation and non-emotional dysregulation as well as the emotional vs. non-emotional aspects of different tasks must be taken into consideration. This has rarely been done in studies of BPD and ASPD. However, some research on CD in children has tried to control for ADHD and even directly test for differences between patients with “pure” CD vs. patients with “pure” ADHD (Rubia, 2011). In a set of executive function tasks (Rubia, 2011), children with ADHD activated PFC less than children with CD, while on a reward task, children with CD showed less activation in OFC than children with ADHD, as expected from the neuropsychological profiles of the two disorders.

The balance between emotional and non-emotional processes may also be discussed in relation to specific behaviors that are altered in a set of psychiatric states. For example, impulsivity (measured with self-rating questionnaires or behavioral tests such as stop signal or go-no-go tests) has been studied as a neurocognitive endophenotype marker across disorders (Robbins et al., 2012). This suggests that a common underlying process related to impulse control capacity could underlie different disorders. However, impulse control is not a simply unitary construct (Robbins et al., 2012; Sebastian et al., 2014). For example, in BPD, impulsivity is associated with emotional distress and self-injurious behavior while it is associated with both emotional and non-emotional behaviors in ADHD (Sebastian et al., 2014). Thus, impulse control and impulsivity may exist in both non-emotional and emotional domains and represent different cognitive core processes.

Even in the same cognitive task, different components may fail in individuals with non-emotional dysregulation as compared to individuals with emotional dysregulation. Evidence for this was observed in the Imagen project (Schumann et al., 2010) in which almost 2000 children underwent structural and functional imaging scanning and a battery of cognitive tests and clinical questionnaires. In a functional imaging analysis of the stop-signal task, two main regressors (indicating different phenotypes) were tested separately while controlling for the other. One regressor involved misuse of substances as an indicator of [emotional] impulsivity (associated with emotional dysregulation; *n* = 1593) and the other regressor quantified non-emotional ADHD traits (attentional problems and impulsivity; *n* = 342; Whelan et al., 2012). While there was no behavioral difference in performance on the stop-signal test, the substance abuse regressor was associated with hypoactivation in the lOFC during the stop-success aspect of the trial while the ADHD-associated regressor was linked to hypoactivation of bilateral inferior frontal network and BG network during the stop-fail aspects of the trials. Thus, this study suggests that different strategies are used by subjects with emotional dysregulation tendencies vs. non-emotional attentional dysregulation, possibly mirroring an imbalance in the emotional vs. non-emotional regulatory systems.

## A Division Between Emotional and Non-Emotional Regulation Beyond Prefrontal and Cingulate Cortex

### Neuronal Circuitry—Prefrontal-Basal Ganglia Loops

The PFC and ACC work in close connection with the BG and specific neuromodulatory systems, including the dopamine system, to accomplish cognitive computations such as choosing specific behavioral responses, learning reward associations and behaviors, and transforming new behaviors into habits (Graybiel, 2008; Haber and Knutson, 2010; Haber and Behrens, 2014). If emotional and non-emotional processing can be partially differentiated, then so should prefrontal interactions with BG and neuromodulatory systems. In line with this idea, the distinction between OFC/rACC (including vmPFC) and dlPFC/cACC is mirrored in the circuits linking PFC/ACC with BG (Graybiel, 2008; Haber and Knutson, 2010; Haber and Behrens, 2014). The PFC-BG-thalamus-PFC circuit is organized as parallel loops that are both segregated and integrated (Graybiel, 2008; Haber and Knutson, 2010; Haber and Behrens, 2014). While rACC/vmPFC and OFC are particularly strongly connected with ventral striatum, the cACC and dlPFC are strongly connected with dorsal striatum in a graded manner (Graybiel, 2008; Haber and Knutson, 2010; Haber and Behrens, 2014). This allows a degree of separation between reward processes, non-reward cognitive processes and motor processes. As the loops interconnect, the different dimensions can also interact with each other, as is essential. In this way, this system allows a transformation from reward guided responses and reward learning to behavioral habits and compulsions (Graybiel, 2008; Robbins et al., 2012).

### Neuromodulatory Effects of Dopamine and Reward Processing

**Figure 10:**
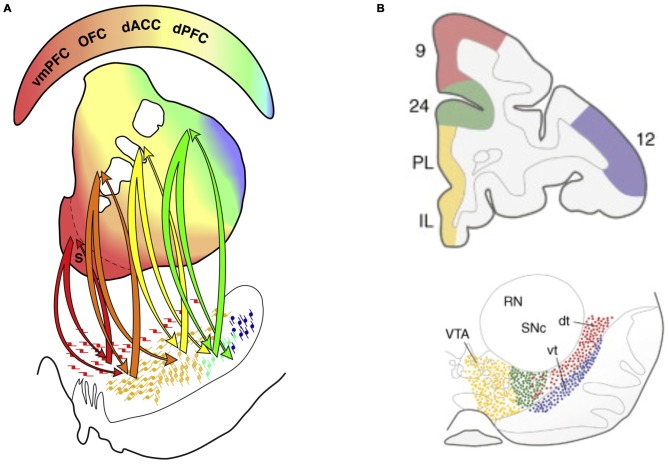
**Connectivity between the dopamine system and basal ganglia (BG), and between the dopamine system and PFC. (A)** The division between different prefrontal and anterior cingulate cortex systems is mirrored in BG organization and dopamine system projections to BG. Color-coded connectivity is shown between dopamine neurons in the midbrain (lower part of the figure) and different parts of the BG (middle part of the figure; the ventral striatum in is shown in red and dorsal striatum is mainly shown in yellow and green) (Haber and Behrens, 2014). The color-coded upper part of the figure indicates that while vmPFC/rACC and orbitofrontal cortex are more connected to the ventral striatum, caudal ACC (cACC, here designated dACC) and dorsal PFC (dPFC) are more connected to the dorsal striatum. **(B)** In primates a distinction between different prefrontal and anterior cingulate cortex areas is also preserved in the dopamine system projecting directly to the cortex (Williams and Goldman-Rakic, 1998; Björklund and Dunnett, 2007). Panel **A** is originally from Haber and Behrens (2014) and reprinted with permission from Elsevier. Panel **B** is originally from Björklund and Dunnett (2007) and reprinted with permission from Elsevier.

The dopamine system is especially interesting in relation to ADHD as it is altered in ADHD and the main pharmacological treatments modulate catecholamines, including dopamine (Swanson et al., 2007, 2011; Volkow et al., 2009). A similar division between an “emotional” and a “non-emotional” dimension can also be discerned in the dopamine system as for the prefrontal and BG circuits (Williams and Goldman-Rakic, 1998; Björklund and Dunnett, 2007; Haber and Behrens, 2014; Figures 10A,B). Dopamine neurons are organized as tiers of nuclei from the ventral tegmental area (VTA) to the substantia nigra. While VTA neurons tend to reach ventral striatum and the subgenual/rostral ACC (including vmPFC), substantia nigra neurons tend to project to the dorsal striatum and the dlPFC—also in a graded manner. The described system allows for both segregated and integrated processing at multiple levels (midbrain/dopamine, BG and PFC/ACC) and is likely relevant to specific behaviors related to ADHD and emotional instability disorders, respectively.

Both associative and instrumental reward learning are dependent on the ventral striatum, which signals reward prediction errors via the dopamine system (Schultz et al., 1997; Schultz, 2007). Functional imaging studies on humans support this model (O’Doherty et al., 2003, 2006; Pessiglione et al., 2006). The reward anticipation period is an important component of this reward learning system (Haber and Knutson, 2010).

Numerous functional imaging studies have found that ADHD patients show a decreased BOLD response in the ventral striatum during the anticipation of rewards (Scheres et al., 2007; Strohle et al., 2008; Stark et al., 2011; Carmona et al., 2012; Edel et al., 2013; Furukawa et al., 2014; Kappel et al., 2015)—making this one of the most robust paradigms in functional imaging research on ADHD—but see von Rhein et al. (2015) and the following discussion (Plichta and Scheres, 2015). Interestingly, even the degree of subclinical ADHD symptoms has been shown to correlate with striatal hypoactivity during reward anticipation in a non-clinical sample (Stark et al., 2011). At the same time, some studies have found a stronger response to reward outcome either in striatum (Furukawa et al., 2014; von Rhein et al., 2015) or in the OFC (Strohle et al., 2008) in ADHD patients vs. controls. Together with hypofunction during reward anticipation, this suggests that ADHD patients are more affected by immediate rewards than by future rewards. Although a robust finding, the striatal reward anticipation dysfunction observed in ADHD patients must remain tentative as emotional instability traits have not been controlled for in most existing ADHD studies. We speculate that the emotional dimension of ADHD will be more related to the differential processing of reward anticipation than the non-emotional dimensions. Few studies have investigated this issue in emotional instability disorders but initial studies show similar results in BPD and ASPD (Vollm et al., 2007).

The decreased striatal BOLD signal in reward anticipation observed in ADHD may be associated with the abnormalities in delay aversion and temporal discounting that are often observed in ADHD (Castellanos et al., 2006; Thorell, 2007; Yu et al., 2015)—as all are related to processing of future rewards (vs. processing an immediate reward). These processes involve error signals from dopamine neurons in the VTA (Haber and Knutson, 2010), also pointing towards dysfunction in the emotional domain. In line with this reasoning, initial studies have shown that emotional instability in CD is related to increased temporal discounting unrelated to ADHD (White et al., 2014). It would therefore be important to test how reward anticipation relates specifically to the emotional vs. non-emotional dimensions in ADHD. Interestingly, timing deficits are highly problematic in non-emotional tasks in patients with ADHD (Rubia et al., 2009) and may be more related to the “non-emotional” dysregulation traits.

Initial studies suggest that the hypoactivation of ventral striatum observed in reward anticipation is more expressed in inattentive ADHD compared to combined type ADHD in adults (Edel et al., 2013) suggesting that sub-categorization of ADHD may be important in understanding how future rewards are processed. In line with this, non-emotional trait impulsivity in the general population is related to increased striatal activation in the reward anticipation phase in contrast to hypoactivation in ADHD (Plichta and Scheres, 2014). Different models have been evoked to explain this discrepancy (Plichta and Scheres, 2014). However, an unmentioned possibility is that emotional dysregulation may be linked to striatal hypoactivation in ADHD but not to the trait-impulsivity of healthy subjects. This would be in line with the finding that subclinical ADHD symptoms are also associated with lower striatal activation during reward anticipation (Stark et al., 2011). Thus, non-emotional trait impulsivity and ADHD symptoms appear to be dissociated in the general population in relation to reward processing.

## Interoception Vs. Exteroception as an Alternative Framework

The differentiation of information processing into an “emotional” domain and a “non-emotional” domain may be criticized in several ways. First, the definition of emotion is at best weak. Emotion pertains to a subjective experience. However, many associated processes that deal with threats, rewards, or social signals are subconscious—but are also referred to as emotional processes. Moreover, some fast behavioral responses that are associated with threat or reward cues that may start even before the emotional experience are also termed emotional responses.

The common theme with all “emotional” processes is that they are directly or indirectly associated with interoceptive systems. While exteroceptive stimuli involve the external world, interoceptive stimuli relate to the state of the body, its needs and threats (Craig, 2002, 2009). Craig (2002, 2009) has suggested that interoceptive information from the body directly reaches posterior insula and cACC. Craig (2002, 2009) views the posterior insula as the primary cortex for interoceptive stimuli (“primary interoceptive representation”), and the cACC as a limbic motor region. The interoceptive signal progresses forward in the insula and is integrated with information from various sensory modalities (via, for example, higher-order sensory regions, the temporal pole and the amygdala) and prefrontal input (including information from OFC) to form a “meta-representation” in the anterior insula. According to this theory, such meta-representations form the moment-to-moment emotional states that we experience subjectively (termed “global emotional moments”). These representations are considered fundamental for emotional experiences.

Apart from interoceptive signals from the body being re-represented to construct a subjective feeling state, emotional states may be produced by top-down mechanisms that directly activate emotional processes in anterior insula and ACC. Similarly, exteroceptive signals may trigger conditioned responses in these systems. It could be argued that such top-down induced processes are closely associated to the interoceptive system by analogy with visual imagination induced by top-down systems. For example, increased activity in anterior insula and ACC in empathy for pain overlaps with the activations induced by nociceptive signals associated with the experience of pain (Singer et al., 2004). The extent to which the top-down induced activity in empathy for pain is related to the processing of bottom-up nociceptive input is under debate (Singer et al., 2004; Wager et al., 2013; Rutgen et al., 2015).

Top-down activated emotional processes may be also indirectly associated to interoceptive systems through the activation of bodily responses and induction of peripheral perceptions (e.g., through emotional expressions and autonomic responses), which in turn can provide interoceptive feedback to “hot” circuits (James, 1890; Damasio, 1993; Craig, 2002, 2009). Thus, there is a tight relation between bottom-up and top-down influences on emotional processes.

While it is not clear to what extent early interoceptive signals are prone to modulation, it has been shown that all parts of insula (posterior, mid and anterior) as well as cACC may be regulated by top-down systems during processing of bottom-up interoceptive input (Atlas and Wager, 2014) and processing of top-down induced activation in the interoceptive stream (Singer et al., 2006; Rutgen et al., 2015).

Apart from insula and ACC, several subcortical regions are important for processing stimuli that are closely associated to the bodily state. External signals related to threat and rewards are not directly generated in the interoceptive environment although they are of fundamental importance for survival. Such signals are dependent on amygdala and ventral striatum, sub-cortical regions that often are co-activated with insula and ACC (Paulus et al., 2005; Paulus and Stein, 2006; Kable and Glimcher, 2007; Milad and Quirk, 2012). Thus, amygdala and ventral striatum form a tight network with cortical interoceptive structures. Apart from a direct interaction with insula and ACC, signals from amygdala and ventral striatum may also re-enter the interoceptive loop through proprioceptive feedback from behavior and autonomic responses related to fear and reward. Therefore, the processing of certain external inputs in these structures, including threats and rewards, may be viewed as highly associated to the interoceptive dimension.

Given the difficulties of differentiating “emotional” and “non-emotional” processing, an alternative is a division into one set of processes related only to the “exteroceptive” dimension and another set of processes associated to the “interoceptive” dimension focused on bodily survival and homeostasis. Importantly, even in this type of division of information processing, we note that exteroceptive information interacts with interoceptive information at many stages of the brain hierarchies.

The division between a network that is mostly focused on exteroceptive processes and a network that is associated with interoceptive processes (such as body states and the needs of the bodily functions related to survival) suggests a demand for two different regulatory functions as well. One proposal would be that cACC and dlPFC regulate processes related to the exteroceptive world, while rACC and lOFC regulate processes related to the interoceptive world and its needs by controlling information processing in insula, cACC, amygdala, ventral striatum and the brainstem. As every individual acts in an external environment, these networks need to interact at multiple levels. The division between interoceptive and exteroceptive networks, including their specific top-down regulatory systems, fits with the observation that some individuals have problems that relate more to a specific dimension of top-down control such as “non-emotional” regulation or “emotional” regulation. A testable hypothesis derived from this reasoning is whether processing of more classical interoceptive input is also dysregulated in subjects with emotional instability.

## An Integrative Model of ADHD and Emotional Instability

We have presented a model in which ADHD and emotional instability disorders (such as BPD, ASPD and CD) are mechanistically related in that they all involve similar dysfunctional top-down regulation of information processing. We hypothesized that the difference between the classically defined disorders (ADHD vs. emotional instability disorders) is whether this dysfunctional regulation is related to emotional (and interoceptively associated) processing or non-emotional (and exteroceptively associated) processing (*Hypothesis 1*).

To probe this hypothesis, we have shown that it is possible to divide these two dimensions of top-down control. In summary, we have discussed that information processing in the brain is highly dependent on complex reciprocal interactions between multiple regions in large-scale networks (Mesulam, 1998, 2012; Engel et al., 2001; Dehaene and Changeux, 2011; Siegel et al., 2012) rather than isolated processes in specific regions (such as specific emotional and non-emotional regions). Moreover, emotional and non-emotional networks cannot be rigidly differentiated—since most tasks require subcomponents that include both types of processes and information interacts at every hierarchical stage. Finally, some structures may perform information processing that pertains both to emotional and non-emotional processes (Shackman et al., 2011). Nevertheless, the clinical perspective suggests that some specificity must exist, indicating that different networks focus on either emotional or non-emotional processing that can be separated to a certain degree. It is likely that each of these two putative networks has a hierarchical perception-action organization (Fuster, 2000, 2004; Fuster and Bressler, 2012), where the highest levels process more complex and temporally dispersed information. In line with this view, we have reviewed research suggesting that cACC and dlPFC perform similar types of information processing for the non-emotional (and exteroceptive) processing stream, as rACC and lOFC perform for the emotional (and interoceptive) processing stream. Both top-down systems interact with BG to choose and learn behaviors. Moreover, specific parts of the dopamine system interact with the respective system.

While it seems plausible to partially divide top-down regulatory systems that are more specifically tuned to regulate emotional and non-emotional processes, future research must better understand the degree of specificity in these systems by controlling for the other dimension—as few studies have done so far. A prediction would be that cACC and dlPFC should be more involved in top-down regulation of non-emotional processes after controlling for emotional components while rACC and lOFC should be more involved in top-down regulation of emotional processes after controlling for non-emotional components.

Similarly, we have reviewed research suggesting that ADHD involves dysfunction of cACC and dlPFC in non-emotional top-down control while emotional instability disorders involve dysfunction in rACC and lOFC in emotional regulation, in line with the predicted hypothesis. However, these studies often suffer from lack of control of the other dimension, which needs to be addressed in future studies.

**Figure 11:**
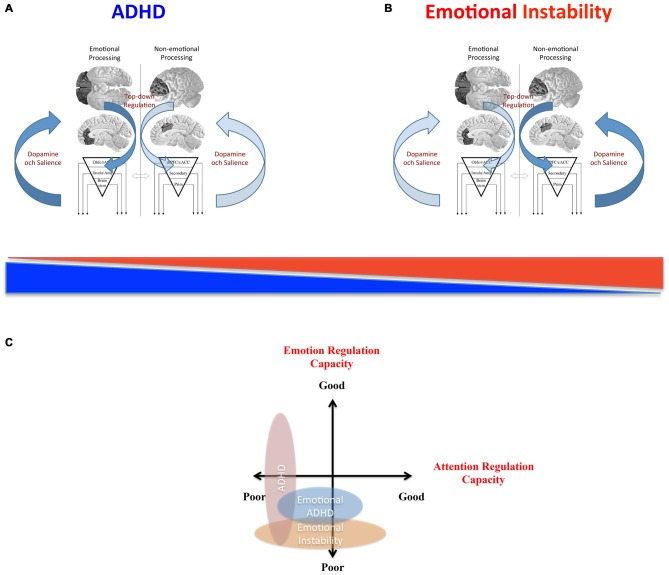
**Model of relation between emotional and non-emotional top-down regulation in ADHD and emotional instability. (A)** In classical ADHD, top-down control of exteroceptive processes subserved by dlPFC and cACC and the associated dopamine system and dorsal striatum are dysfunctional (affecting “cool” executive functions)—shown in light blue. **(B)** In emotional instability disorders [such as borderline personality disorder (BPD), conduct disorder (CD) and antisocial personality disorder (ASPD)], the top-down control of interoceptive and emotional processes induced by orbitofrontal cortex OFC and rostral ACC and associated dopamine system and ventral striatum are dysfunctional (affecting “hot” executive functions and emotional regulation)—shown in light blue. Instead of representing two extreme variants, dysregulation in different individuals lies on a gradient extending from mostly non-emotional top-down control (all blue) to mainly emotional top-down control (all red). **(C)** A two-dimensional model indicating “non-emotional” attention regulation capacity on one axis and “emotional” regulation capacity on the other axis can incorporate ADHD, emotional ADHD and emotional instability disorders such as BPD, ASPD and CD.

A related hypothesis predicts that the classically defined phenotypes of “pure” ADHD and emotional instability disorders should be unusual if the underlying processes are mechanistically related—instead most patients will have both components in different proportions (*Hypothesis 2*). Therefore, a dimensional approach should better describe the problems of most patients. In the present article we have highlighted research suggesting that top-down regulatory capacities of the emotional and the non-emotional systems vary in their efficiency among subjects in the general population (Das et al., 2012; Petrovic et al., 2015). An extremely poor cognitive core capacity in such top-down regulation may be described as a dysregulation since it would be associated with clinical symptoms and functional loss. Dysregulation strictly encompassing the non-emotional (and exteroceptive) processes would be related to ADHD, while dysregulation strictly pertaining to the emotional (and interoceptive) processes would be related to emotional instability disorders (Figures 11A,B). It would therefore be possible to describe both types of disorders on a two dimensional scale where poor capacity for non-emotional regulation but normal (or at least not handicapping) emotional regulation would be associated with ADHD (Figure 11C). On the same scale, poor capacity for emotional regulation but normal (or at least not handicapping) non-emotional regulation would be associated with emotional instability disorders such as BPD, ASPD or CD. Comorbid states would have poor capacity for both non-emotional and emotional processing. Emotional ADHD would be explained as a disorder in which there is dysregulation of both non-emotional and emotional processes (but to a milder degree). However, these dysregulatory capacities are not independent. As we discussed previously, there is a substantial overlap between the disorders and a tight relation between degree of symptoms both in patients and in healthy individuals in line with a common underlying mechanism for all these clinical states. Possibly, the best way to describe these clinical states is with traits related to emotional dysregulation and traits related to non-emotional dysregulation that are combined to different degrees.

One possible primary candidate for a common substrate that may incorporate both dimensions is the dopamine system. This neuromodulatory system is implicated in ADHD (Swanson et al., 2007; Volkow et al., 2009) and may affect both emotional and non-emotional processing. The possibility that the dopamine system may be similarly altered in both ADHD and emotional instability disorders suggests that the same treatment, i.e., dopaminergic agonists, may be efficacious for both categories of disorders (*Hypothesis 3*). This idea is in line with initial studies, suggesting that methylphenidate also has an impact on emotional dysregulation behaviors in ASPD and CD (Kaplan et al., 1990; Brown et al., 1991; Klein et al., 1997; Connor et al., 2000) as well as BPD (Schulz et al., 1988; Golubchik et al., 2008; Prada et al., 2015; although see Schulz et al. (1988) for possible issue with schizotypal comorbidity). Interestingly, central stimulants also reduce behaviors related to emotional impulsivity and instability in ADHD such as suicide rate (Chen et al., 2014), drug use (Chang et al., 2014) and criminal behavior (Lichtenstein et al., 2012)—possibly indicating a specific effect on emotional dysregulation in ADHD. One possibility is that there may be graded variability in capacity of the dopamine system in the ventral vs. dorsal tiers of dopamine neurons. This would then be mirrored in and impact the whole regulatory brain network including BG, ACC and PFC processing either emotional or non-emotional related information. It would affect higher order processing and regulation as well as brain structure on a longer term.

Here, we have argued that there is a mechanistic relation between ADHD and emotional instability disorders such as BPD, ASPD and CD. Perhaps emotional instability disorders should not be regarded as personality disorders but as emotional neuropsychiatric states. Also, using a dimensional approach, characteristics of patients may be better described than using standard categorical distinctions among psychiatric disorders. This may both improve understanding of patients’ needs and their treatment. However, the model presented here is theoretical, and must be tested and scrutinized in detail. To better understand how ADHD relates to emotional instability disorders, traits must be measured in patients and healthy participants and experimental studies must be designed that can control for both non-emotional and emotional components of different processes.

## Author Contributions

PP and FXC wrote the article. Both authors have made substantial, direct and intellectual contribution to the work, and approved it for publication.

## Funding

PP was funded by Swedish Research Council “Vetenskapsrådet” (K2009-61P-21306-04-4) and “Projektmedel för forskning och utveckling inom psykiatri, primärvård och geriatrik, PPG” (20140301). FXC was partially supported by NIH grant U01MH099059.

## Conflict of Interest Statement

The authors declare that the research was conducted in the absence of any commercial or financial relationships that could be construed as a potential conflict of interest.
